# Multitask prediction models for serous ovarian cancer by preoperative CT image assessments based on radiomics

**DOI:** 10.3389/fmed.2024.1334062

**Published:** 2024-02-06

**Authors:** Le Fu, Wenjing Wang, Lingling Lin, Feng Gao, Jiani Yang, Yunyun Lv, Ruiqiu Ge, Meixuan Wu, Lei Chen, Aie Liu, Enhui Xin, Jianli Yu, Jiejun Cheng, Yu Wang

**Affiliations:** ^1^Department of Radiology, Shanghai First Maternity and Infant Hospital, School of Medicine, Tongji University, Shanghai, China; ^2^Department of Obstetrics and Gynecology, Shanghai First Maternity and Infant Hospital, School of Medicine, Tongji University, Shanghai, China; ^3^Shanghai United Imaging Intelligence Co., Ltd., Shanghai, China

**Keywords:** radiomics, preoperative evaluation, serous ovarian cancer, computer tomography, nomogram

## Abstract

**Objective:**

High-grade serous ovarian cancer (HGSOC) has the highest mortality rate among female reproductive system tumors. Accurate preoperative assessment is crucial for treatment planning. This study aims to develop multitask prediction models for HGSOC using radiomics analysis based on preoperative CT images.

**Methods:**

This study enrolled 112 patients diagnosed with HGSOC. Laboratory findings, including serum levels of CA125, HE-4, and NLR, were collected. Radiomic features were extracted from manually delineated ROI on CT images by two radiologists. Classification models were developed using selected optimal feature sets to predict R0 resection, lymph node invasion, and distant metastasis status. Model evaluation was conducted by quantifying receiver operating curves (ROC), calculating the area under the curve (AUC), De Long’s test.

**Results:**

The radiomics models applied to CT images demonstrated superior performance in the testing set compared to the clinical models. The area under the curve (AUC) values for the combined model in predicting R0 resection were 0.913 and 0.881 in the training and testing datasets, respectively. De Long’s test indicated significant differences between the combined and clinical models in the testing set (*p* = 0.003). For predicting lymph node invasion, the AUCs of the combined model were 0.868 and 0.800 in the training and testing datasets, respectively. The results also revealed significant differences between the combined and clinical models in the testing set (*p* = 0.002). The combined model for predicting distant metastasis achieved AUCs of 0.872 and 0.796 in the training and test datasets, respectively. The combined model displayed excellent agreement between observed and predicted results in predicting R0 resection, while the radiomics model demonstrated better calibration than both the clinical model and combined model in predicting lymph node invasion and distant metastasis. The decision curve analysis (DCA) for predicting R0 resection favored the combined model over both the clinical and radiomics models, whereas for predicting lymph node invasion and distant metastasis, DCA favored the radiomics model over both the clinical model and combined model.

**Conclusion:**

The identified radiomics signature holds potential value in preoperatively evaluating the R0, lymph node invasion and distant metastasis in patients with HGSC. The radiomics nomogram demonstrated the incremental value of clinical predictors for surgical outcome and metastasis estimation.

## 1 Introduction

High-grade serous ovarian cancer (HGSOC) is the malignant tumor with the highest mortality rate in female reproductive system at present. The early clinical symptoms of HGSOC are not obvious, and most patients are already in the middle to late stage when detected. The current standard treatment methods for HGSOC are platinum-based chemotherapy after primary tumor reduction surgery (PDS), or intermittent tumor reduction surgery after neoadjuvant chemotherapy (1). Studies have shown that these two treatment methods can achieve similar prognosis in patients with stage IIIC-IV ovarian cancer (2). However, for patients who are suitable for early surgical intervention, the biggest risk of undergoing surgical treatment after chemotherapy is the possibility of losing the opportunity for early surgery and developing tolerance to chemical drugs (3). Additionally, the residual lesion size after tumor reduction surgery is one of the most important independent risk factors for the prognosis and survival of ovarian cancer patients (4). Therefore, precise preoperative evaluation of tumors is crucial for selecting treatment plans. Factors such as lymph node invasion, distant metastasis, and whether complete resection of all visible diseases (R0 resection) can be achieved are important considerations for preoperative evaluation.

Radiomics is a powerful and promising image mining method that utilizes high-throughput feature selection based on imaging data. It has been proven to improve diagnostic accuracy, evaluate treatment response, and predict prognosis (5, 6). Several published radiomics prediction models have been established based on computer tomography (CT) (7, 8). However, these radiomics models mainly focused on the location, size, and the metastasis of the abdomen, and they all focused on a single prediction point. In this study, we aimed to establish multitask prediction models for HGSOC by utilizing preoperative CT image assessments based on radiomics.

## 2 Materials and methods

### 2.1 Study population

This retrospective study was approved by our hospital ethics committee, and the requirement for patient informed consent was waived. A total of 112 consecutive patients (age range: 36–84 years) with confirmed serous ovarian cancer based on pathology were enrolled in our study. The enrollment period spanned from November 2012 to January 2022.

Patients’ laboratory findings, including serum cancer antigen-125 (CA125), serum human epididymis protein 4 (HE-4) level, and neutrophil-to-lymphocyte ratio (NLR), were collected from the electronic medical record. Additionally, preoperative unenhanced CT scans of the abdomen and pelvis were obtained. A total of 112 patients were included in the study to predict R0 status, lymph node invasion, and distant metastasis.

### 2.2 Radiomics analysis

The workflow of radiomics analysis consists of five steps: obtaining ROI, computing features, selecting features, constructing the model, and evaluation. Radiomics analysis was performed using the uAI Research Portal (United Imaging Intelligence, China), which is a clinical research platform implemented in Python programming language (version 3.7.3). the widely used package PyRadiomics package^1^ (9, 10) was utilized for this analysis.

**Figure 1:**
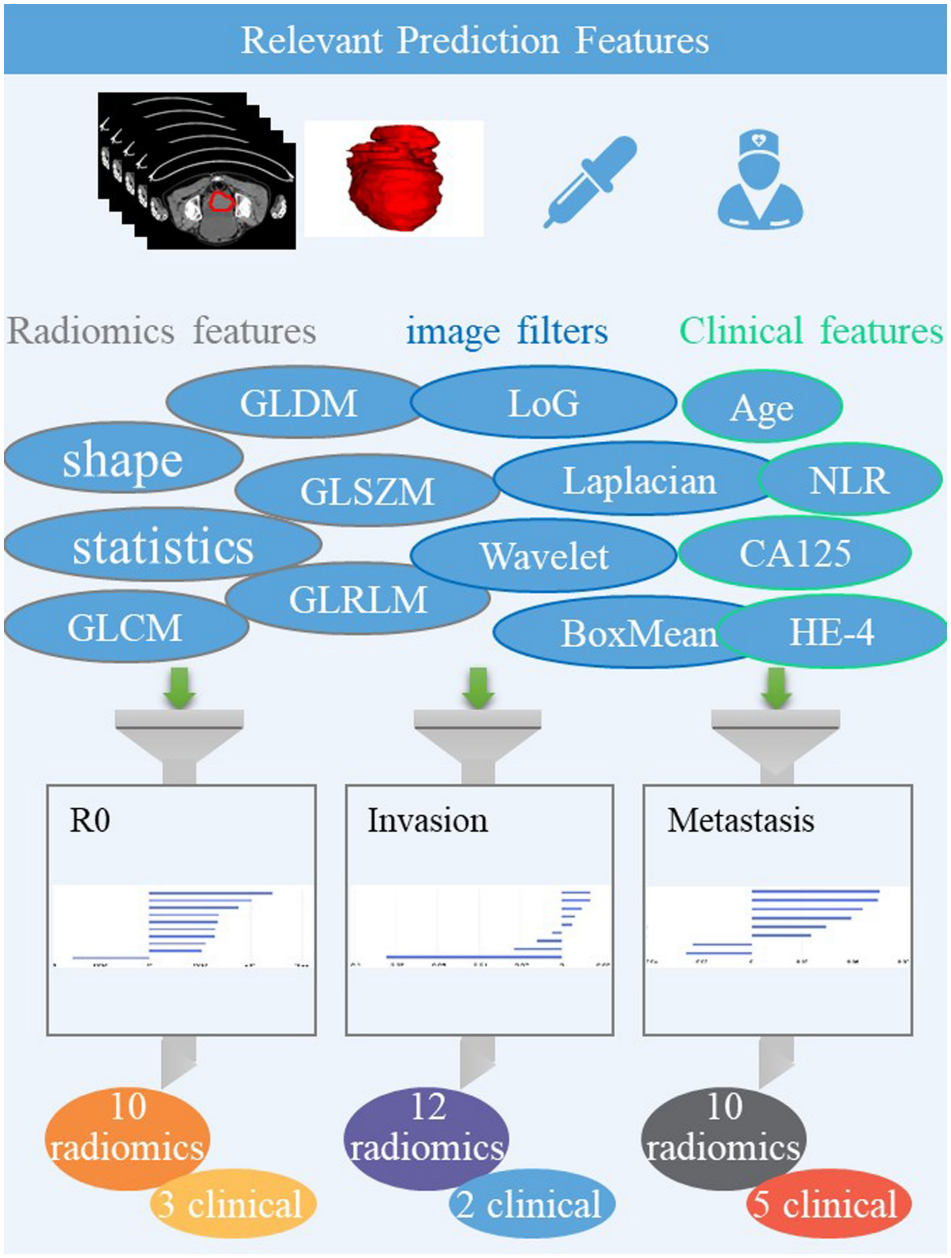
Flowchart of radiomics analysis.

The volume of the entire ovarian lesion was manually delineated on CT images by two radiologists (L. Fu with 12 years of imaging experience and WJ. Wang with 14 years of imaging experience) using the uAI Research Portal, denoted as ROI (region of interest). A total of 2,264 radiomic features were extracted from the ROI on each CT image, including 104 original features grouped as: 18 the first-order statistics, 72 texture, and 14 shape features. Among 14 shape features, selection was done only on original images, while the others were based on both original images and images processed through 25 filters such as boxmean, wavelet, laplacian, etc. To account for any difference in index dimension, the extracted radiomic features for each sequence were standardized into normal distributed z-scores. For the three classification tasks, the top 10, 9, and 8 highest-ranking radiomic features were selected, respectively, on CT images using feature selection methods such as K best and least absolute shrinkage and selection operator regression (LASSO), as shown in Figure 1.

To evaluate the performance of the classifier and protect against overfitting due to the limited amount of data, we used the cross-validation method. Specifically, we employed 5-fold cross-validation (9). The feature set was randomly split into five partitions, ensuring that each partition maintained the same ratio of positive and negative images. During each fold, the classifier was trained on four-fifths of the dataset and validated on the remaining partition. This process was repeated five times with different subgroups, resulting in five distinct training/testing sets. The average performance across these folds was then calculated to obtain an overall result. To maximize the discrimination ability of the radiomics algorithm, we implemented machine learning classifiers including logistic regression (LR), random forest, decision tree, and support vector machine (SVM) for model construction.

Using the selected optimal feature sets, we constructed classification models to predict R0 status, lymph node invasion, and distant metastasis. Finally, we plotted receiver operating characteristic (ROC) curves and calculated the area under the curve (AUC), sensitivity, specificity, and accuracy to evaluate the performance of the models.

### 2.3 Statistical analysis

All statistical analyses for the present study were performed using SPSS software (version 26.0), R (version 4.1.0), and Python (version 3.5.6). A significance level of *p* < 0.1 was considered statistically significant. DeLong’s test was used to compare the AUC values of different models.

## 3 Results

### 3.1 Clinical characteristics

The clinical baseline data of patients are presented in Table 1 for three postoperative predictors. Firstly, a total of 112 patients were included to predict R0 status, with 80 patients classified as non-R0 status and 32 patients as R0 resection. The cancer antigen-125 (CA125), human epididymis protein 4 (HE-4), and lymphocyte levels showed significant differences between the R0 and non-R0 groups (*p* < 0.05), while no significant differences were observed in patient age, neutrophil-to-lymphocyte ratio (NLR), and Neutrophils (all *p* > 0.05). Secondly, among the 112 patients collected for predicting lymph node invasion, 58 patients had non- Invasion status and 54 patients had Invasion. The NLR and Neutrophils showed significant differences (*p* < 0.05), while no significant difference was detected in other characteristics. Thirdly, the 112 patients were collected to predict distant metastasis, with 79 patients having non- Metastasis status and 33 patients having Metastasis. The age did not show a significant difference (*p* > 0.01), while significant differences were noted in other characteristics (all *p* < 0.05).

**Table 1:** Patient characteristics for three predicting postoperative results.

	Predicting R0			Predicting lymph node invasion			Predicting distant metastasis		
	Non-R0 (*n* = 80)	R0 (*n* = 32)	*p*	Non-invasion (*n* = 58)	Invasion (*n* = 54)	*p*	Non-metastasis (*n* = 79)	Metastasis (*n* = 33)	*p*
Age, mean (SD)	61.7 ± 8.9	60.1 ± 9.9	0.426	61.8 ± 8.5	60.5 ± 9.9	0.475	61.1 ± 9.4	61.3 ± 8.9	0.904
CA125, median (IQR)	880 (415, 2,155)	509 (187, 1774)	0.039	657 (291, 1860)	1,000 (421, 2024)	0.094	635 (288, 1,355)	1,432 (834, 2,283)	0.000
HE-4, median (IQR)	452 (381, 856)	394 (197, 642)	0.013	452 (198, 706)	452 (376, 944)	0.091	452 (220, 643)	642 (404, 1,011)	0.017
NLR, median (IQR)	3.58 (3.0, 5.59)	3.55 (2.71, 4.96)	0.074	3.58 (2.69, 4.67)	4.39 (2.91, 6.48)	0.026	3.58 (2.69, 4.97)	4.62 (3.13, 6.55)	0.002
Neutrophils, median (IQR)	4.70 (3.36, 6.44)	4.43 (4.02, 5.15)	0.282	4.64 (3.08, 5.19)	4.71 (4.08, 6.48)	0.045	4.32 (3.33, 5.58)	4.91 (4.42, 5.80)	0.023
Lymphocyte, median (IQR)	1.23 (0.95, 1.39)	1.42 (1.12, 1.74)	0.007	1.27 (1.07, 1.48)	1.23 (0.86, 1.42)	0.183	1.30 (1.07, 1.50)	1.10 (0.85, 1.30)	0.032

*Negative/positive; *p*-values in bold indicated that the corresponding variables were closely related to the two types in the univariable logistic regression (*p* < 0.1). OR, odd ratio; CI, confidence interval; CA125, cancer antigen-125; HE-4, human epididymis protein 4; NLR, neutrophil-to-lymphocyte ratio.

### 3.2 Performances of clinical and CT radiomics models

The predictive performance of each model is presented in Table 2. The clinical models exhibited relatively poor predictive performance in the testing set, utilizing six clinical characteristics (AUC_R0 = 0.675, AUC_Invasion = 0.591, AUC_Metastasis = 0.729). In contrast, the radiomics models on CT images demonstrated better performance in the testing set (AUC_R0 = 0.872, AUC_Invasion = 0.770, AUC_Metastasis = 0.795) than the clinical models. De Long’s test indicated significant differences between the radiomics and clinical models for predicting R0 and lymph node invasion in the testing set (*p* = 0.003 and 0.011, respectively). However, there was no statistical difference between the radiomics and clinical models for predicting distant metastasis (*p* = 0.367).

**Table 2:** The 5-fold mean performance of the clinical model, radiomics model and radiomics + clinical model for predicting postoperative results.

	Model	AUC	Sensitivity	Specificity	Accuracy
Predicting R0	Clinical	0.675 (0.567–0.784)	0.652	0.648	0.649
	Radiomics	0.872 (0.806–0.938)	0.819	0.835	0.827
	Radiomics+ Clinical	**0.881** (0.806–0.956)	0.833	0.784	0.802
Predicting	Clinical	0.591 (0.406–0.885)	0.647	0.489	0.563
Lymph node	Radiomics	0.770 (0.563–0.972)	0.704	0.702	0.705
Invasion	Radiomics+ Clinical	0.800 (0.614–0.970)	0.776	0.689	0.732
Predicting	Clinical	0.729 (0.425–0.925)	0.686	0.697	0.696
Distant	Radiomics	0.795 (0.572–0.998)	0.729	0.718	0.722
Metastasis	Radiomics+ Clinical	0.796 (0.623–0.981)	0.781	0.711	0.731

The highest AUC was bold in Table 2.

### 3.3 Performances of radiomics-clinical comprehensive models

The model for predicting R0 was developed using 10 radiomics features and three clinical features, including CA125, HE-4, lymphocyte. The AUCs of the combined model were 0.913 and 0.881 in the training and testing datasets, respectively (Figure 1 and Table 2). De Long’s test revealed significant differences between the combined and clinical models in the testing set (*p* = 0.003). However, there was no statistical difference between the combined and radiomics models (*p* = 0.756).

The predicting lymph node invasion model was established based on 12 radiomics features and two clinical features, including NLR and Neutrophils. In the training and test datasets, the combined model achieved AUCs of 0.868 and 0.800, respectively. Significantly different results were observed between the combined and clinical models in the testing set according to De Long’s test (*p* = 0.002). However, no statistical difference was found between the combined and radiomics models (*p* = 0.185).

In establishing the predicting distant metastasis model, we utilized 10 radiomics features and five clinical features, including CA125, HE-4, NLR, Neutrophils, and lymphocyte. The combined model achieved AUCs of 0.872 and 0.796 in the training and test datasets, respectively. However, there was no statistical difference observed among the three models, as all *p*-values were greater than 0.05.

### 3.4 The calibration and clinical utility of all models

**Figure 2:**
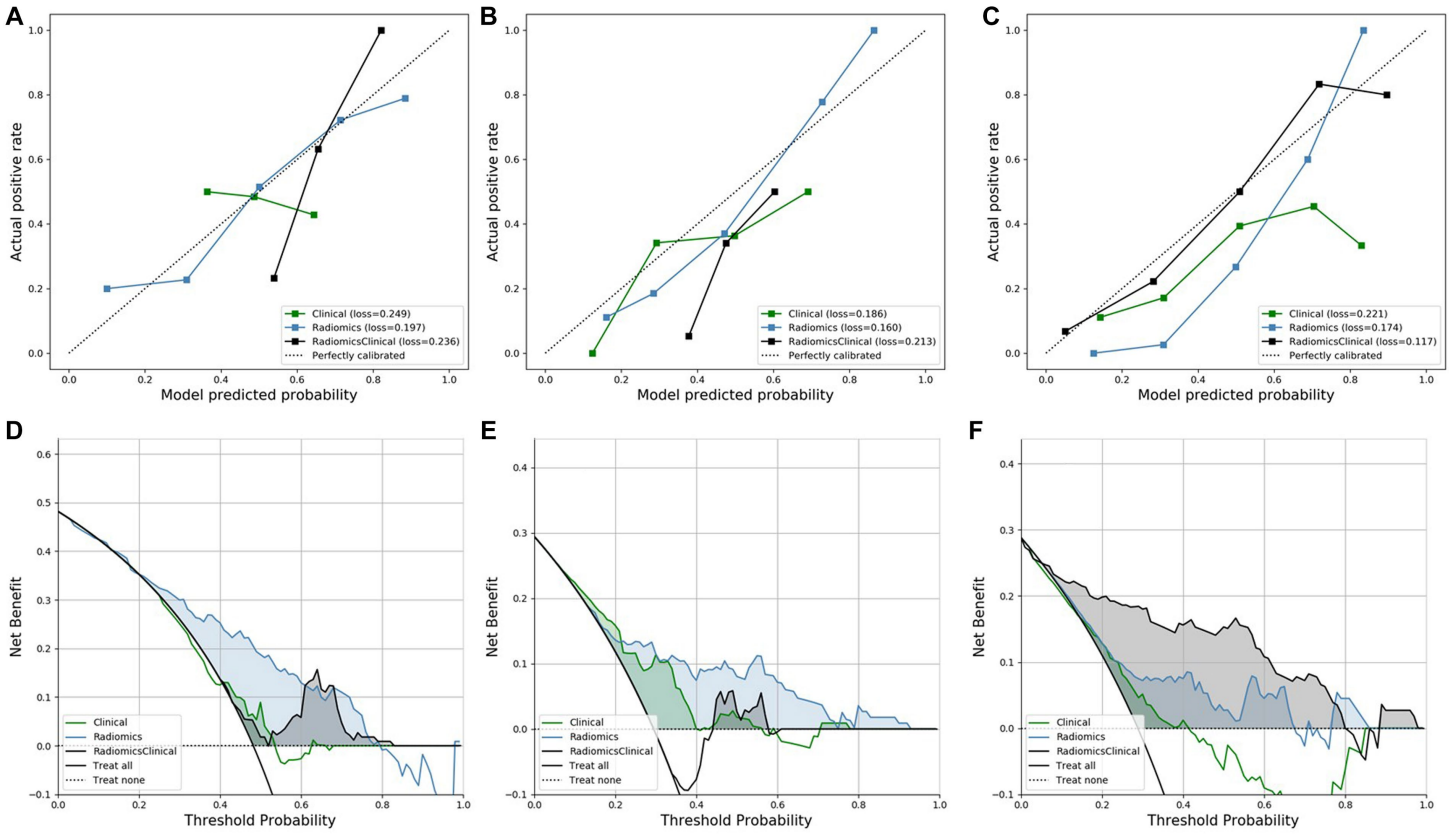
The calibration curves and DCA curves of all models. **(A–C)** The calibration curves for predicting lymph node invasion, distant metastasis, and R0, respectively. **(D–F)** The DCA curves for predicting lymph node invasion, distant metastasis, and R0, respectively.

The calibration curves of all models are shown in Figures 2A–C. The calibration of the radiomics model for predicting lymph node invasion was superior to that of the clinical model and combined model. Similarly, the calibration of the radiomics model for predicting distant metastasis outperformed that of the clinical model and combined model. On the other hand, the combined model for predicting R0 demonstrated excellent agreement between the observed and predicted results, surpassing both the clinical model and radiomics model.

The DCA of all models is displayed in Figures 2D–F, illustrating the clinical utility. The DCA of the radiomics model for predicting lymph node invasion outperformed the clinical model and combined model. Furthermore, the DCA of the radiomics model for predicting distant metastasis exhibited better results compared to both the clinical model and combined model. Similarly, the DCA of the combined model for predicting R0 demonstrated superior performance compared to both the clinical model and radiomics model.

## 4 Discussion

Accurate and non-invasive prediction of R0, lymph node invasion, and distant metastasis is crucial for implementing individualized management and improving the prognosis of patients with HGSOC. In this study, we developed and validated three multitask prediction models for HGSOC that integrate CT radiomics features and clinical information. The combined model demonstrated excellent performance in predicting R0, with AUCs of 0.913 and 0.881 in the training and testing datasets, respectively. For predicting lymph node invasion, the combined model achieved AUCs of 0.807 and 0.800 in the training and testing datasets, respectively. Additionally, for predicting distant metastasis, the combined model achieved AUCs of 0.807 and 0.800 in the training and testing datasets, respectively. Notably, the AUC values of the combined models were consistently higher than those of the clinical models in both the R0 cohort and lymph node invasion cohort.

Most studies have only focused on the features of metastases for R0 prediction (8, 11). The observation of extensive metastases in the abdomen, affected by bowl and ascites, might make it hard to accurately predict R0 in practice. Studies had also shown that the likelihood of metastases can be predicted through the assessment of primary tumors (12, 13). Radiomics has been shown to be a high-performance method for accurately predicting treatment response by assessing tumor heterogeneity (14). Rizzo et al. found that patients with values below the median for F2-Shape/Compactness1, F1- GrayLevelCooccurenceMatrix25/0-1InformationMeasureCorr2, and above the median for F1-GrayLevelCooccurenceMatrix25/−333-1InverseVariance showed a higher risk of residual tumor (36, 36, and 35%, respectively, as opposed to 18, 18, and 18%). However, models were not developed for prediction, or considering the value of clinical information (15). A radiomics signature–based nomogram was developed for the preoperative prediction of R0 in patients with advanced HGSOC. It demonstrated favorable performance in both training and validation sets with an AUC of 0.815 and 0.803, respectively (16). The metastatic situation of HOSG determines the FIOG stages and operation range, actual assessment carries significant weight. The radiomics nomogram demonstrated favorable calibration and discrimination in both the training cohort (AUC = 0.821) and test cohort (AUC = 0.843) (17). Previous studies on radiomic for predicting R0, lymph node invasion and distant metastasis have been based on CT enhanced images and MR enhanced images. However, some individuals may be allergic to contrast agents or have renal dysfunction, making them unsuitable for enhanced CT/MRI examination. Additionally, some individuals may have claustrophobia or metal implants that are not suitable for MRI examination. Therefore, we explored the performance of radiomic based on CT examination without injecting contrast agent in predicting R0, lymph node invasion, and distant metastasis. The three clinical-radiomic combined models showed good performance, indicating that CT examination without contrast agent can provide favorable evidence for the preoperative evaluation of ovarian cancer.

Wavelet features belong to higher-order statistical features, which can more comprehensively reflect the heterogeneity of the original image and may also result in more valuable features than the original image (18). The three prediction models in this study include many Wavelet feature (Figure 1). This suggests that higher-order texture features have a good correlation with surgical outcomes and metastasis in advanced serous cancer patients. A model for predicting platinum resistance was constructed based on the radiomic features proposed by T2WI, DWI, and CE-T1WI sequences of 114 EOCs. In the validation set, the AUC was 0.89 (accuracy = 85.0%, sensitivity = 87.0%, and specificity = 80.0%) (19). The platinum resistance prediction model also includes 9 wavelet features, which is similar to our research results and once again confirms the good performance of wavelet features for preoperative evaluation of advanced serous ovarian cancer.

This study selected clinical features and laboratory examination indicators such as age, CA125, HE4, lymphocytes, neutrophils, and NLR to explore the differences between different groups. The clinical model composed of CA125, HE-4, and lymphocytes showed poor performance. However, with the addition of radiomic features, the performance of the model for predicting R0 significantly increased. The clinical model composed of NLR and neutrophils showed average performance but with the addition of radiomic features, the efficiency of the model for predicting lymph node infiltration significantly increased. This suggests a good correlation between radiomic features and surgical outcomes, as well as lymph node metastasis. The clinical models for predicting distant metastasis composed of CA125, HE-4, NLR, neutrophils, and lymphocyte generally performed good. However, the addition of radiomic features did not significantly improve the performance of the model possibly due to a small sample size. The predictive value of some blood inflammatory composite markers in OC has been extensively reported (20). They can be used for early detection and differential diagnosis of OC and can also predict survival, treatment response, and recurrence in the affected patients. Our results confirmed the NLR is related to R0 status, lymph node invasion, and distant metastasis in HGSC patient. This suggests a close correlation between NLR and surgical outcomes as well as metastasis. Further exploration of these correlations will be conducted at the molecular level in future studies. A radiomic-clinical nomogram based on MRI for predicting R0 also included CA125, LDH, and NLR (16).

The DCA of the combined model for predicting R0 confirmed the incremental clinical utility of the proposed model for individualized prediction. This finding is consistent with several previous radiomics studies on HGSOC and cervical carcinoma (21–23). However, the DCA of the combined models for predicting lymph node invasion and distant metastasis did not show the superior performance compared to a single model. The combined models were less sensitive in evaluating tumor heterogeneity, and other artificial intelligence methods will be explored to optimize the model fusion.

This study had several limitations. First, selection bias was inevitable due to the retrospective nature of the study and strict inclusion and exclusion criteria. Second, the retrospective datasets were relatively small, with an unbalanced distribution of patients. Third, radiomics has inevitable limitations in terms of reproducible application as it heavily relies on artificial segmentation and handcrafted features (24). Moreover, although volumetric tumor segmentation can provide a robust way to characterize tumor heterogeneity, it may be time-consuming, especially for larger ovarian tumors.

## 5 Conclusion

The identified radiomics signature holds potential value in preoperatively evaluating the R0, lymph node invasion, and distant metastasis in patients with HGSC. The radiomics nomogram demonstrated the incremental value of clinical predictors for surgical outcomes and metastasis estimation. However, further external validation is required before its wide clinical application.

## Data availability statement

The raw data supporting the conclusions of this article will be made available by the authors, without undue reservation.

## Ethics statement

This study was approved by the Ethics Committee of the Shanghai First Maternity and Infant Hospital (registration number:KS22281). The studies were conducted in accordance with the local legislation and institutional requirements. The ethics committee/institutional review board waived the requirement of written informed consent for participation from the participants or the participants’ legal guardians/next of kin because This is a retrospective study.

## Author contributions

LF: Conceptualization, Methodology, Writing – original draft. WW: Methodology, Writing – original draft. LL: Formal analysis, Writing – original draft. FG: Writing – review & editing. JYa: Writing – review & editing, Data curation. YL: Writing – review & editing. RG: Writing – review & editing. MM: Writing – review & editing. LC: Writing – review & editing. AL: Writing – review & editing. EX: Writing – review & editing. JYu: Writing – review & editing. JC: Funding acquisition, Writing – review & editing. YW: Writing – original draft.

## Footnotes

^1^ https://pyradiomics.Readthedocs.io/en/latest/index.html
